# Wie hat sich die Lebenserwartung ohne funktionelle Einschränkungen in Deutschland entwickelt? Eine Analyse mit Daten des Deutschen Alterssurveys (DEAS)

**DOI:** 10.1007/s00103-024-03875-9

**Published:** 2024-04-22

**Authors:** Sonja Nowossadeck, Enno Nowossadeck, Fabian Tetzlaff, Juliane Tetzlaff

**Affiliations:** 1 Deutsches Zentrum für Altersfragen, Manfred-von Richthofen-Str. 2, 12101 Berlin, Deutschland; 2 Robert Koch-Institut, Berlin, Deutschland; 3 Medizinische Hochschule Hannover, Hannover, Deutschland

**Keywords:** Behinderungsfreie Lebenserwartung (DFLE), Morbiditätskompression, Funktionelle Einschränkungen, Global Activity Limitation Indicator (GALI), Mobilitätseinschränkungen, Disability-free life expectancy (DFLE), Compression of morbidity, Functional limitations, Global activity limitation indicator (GALI), Mobility limitations

## Abstract

**Einleitung:**

Der langfristige Anstieg der Lebenserwartung wirft die Frage auf, ob die gewonnene Lebenszeit mit einer Verlängerung der Jahre ohne gesundheitliche Einschränkungen einhergeht. Die Studie untersucht, wie sich die Lebenserwartung ohne funktionelle und Mobilitätseinschränkungen ab dem Alter 46 und 65 Jahre sowie ihre Anteile an der Restlebenserwartung seit 2008 verändert haben.

**Methoden:**

Wir analysieren Daten des Deutschen Alterssurveys der Wellen 2008, 2014 und 2020/2021. Die Lebenserwartung ohne funktionelle Einschränkungen (Disability Free Life Expectancy – DFLE) wurde mit der Sullivan-Methode berechnet. Untersucht wurden starke funktionelle Einschränkungen mit dem „Global Activity Limitation Indicator“ (GALI) und Einschränkungen der Mobilität (Treppensteigen, mehr als 1 km Gehen).

**Ergebnisse:**

Kompression der Morbidität beim GALI ist bei 46- und 65-jährigen Männern seit 2014 zu beobachten, bei gleichaltrigen Frauen dagegen nicht. Bei der Mobilität zeigen 46- und 65-jährige Männer Tendenzen zur Kompression beim Treppensteigen und 46-jährige Männer beim Gehen von mehr als 1 km seit 2014. Die Werte für Frauen stagnieren für die beiden erstgenannten Indikatoren, aber nicht für 46-jährige Frauen beim Gehen von mehr als 1 km seit 2014.

**Diskussion:**

Unsere Analysen zeigen je nach Indikator, Alter und Geschlecht unterschiedliche Trends der DFLE und lassen keine eindeutige Antwort auf die Frage nach Morbiditätskompression oder -expansion zu. Kompression der Morbidität sehen wir eher bei Männern, Tendenzen der Stagnation oder Expansion dagegen eher bei Frauen. Diese Resultate signalisieren Herausforderungen in der Erhaltung der funktionellen Gesundheit vor allem bei Frauen und weisen auf die Notwendigkeit gezielter Interventionen hin, um die Lebensqualität und die gesunde Lebenserwartung zu verbessern.

**Zusatzmaterial online:**

Zusätzliche Informationen sind in der Online-Version dieses Artikels (10.1007/s00103-024-03875-9) enthalten.

## Abstract (en)

**Introduction:**

The long-term increase in life expectancy raises the question of whether the increased life expectancy is accompanied by an extension of years without health limitations. The study analyzes how life expectancy without functional and mobility limitations from the ages of 46 and 65 and their proportions of remaining life expectancy have changed since 2008.

**Methods:**

We analyze data from the German Ageing Survey of the 2008, 2014, and 2020/21 waves. Life expectancy without functional limitations (disability-free life expectancy—DFLE) was calculated using the Sullivan method. Severe functional limitations (using the Global Activity Limitation Indicator—GALI) and mobility limitations (climbing stairs, walking more than 1 km) were examined.

**Results:**

Compression of morbidity in the GALI has been observed in 46- and 65-year-old men since 2014, but not in women of the same age. In terms of mobility, 46- and 65-year-old men show trends towards compression when climbing stairs and 46-year-old men when walking more than 1 km since 2014. The values for women have stagnated for the first two indicators mentioned, but not for 46-year-old women since 2014 when walking more than 1 km.

**Discussion:**

Our analyses show different trends in DFLE depending on the indicator, age, and gender and do not allow a clear answer to the question of morbidity compression or expansion. We tend to see morbidity compression in men, whereas trends of stagnation or expansion tend to be seen in women. These results signal challenges in maintaining functional health, especially in women, and point to the need for targeted interventions to improve quality of life and healthy life expectancy.

## Einleitung

Deutschland erfährt, wie andere Hocheinkommensstaaten, aufgrund kontinuierlich rückläufiger Mortalität und niedriger Geburtenraten tiefgreifende demografische Veränderungen. Der demografische Wandel führt in Deutschland zu einem zunehmend höheren Anteil älterer Menschen [1]. Chronisch progrediente Erkrankungen bestimmen in der alternden Gesellschaft zunehmend das Morbiditätsspektrum, sie haben in der Vergangenheit einen Großteil der älteren Menschen über weite Phasen ihres Lebens begleitet [2, 3]. Dass die Entwicklung der von Krankheit freien Lebenszeit unterschiedlich verläuft, haben frühere Studien bereits gezeigt [4–9]. Inwiefern das Alter mit zusätzlichen Einschränkungen verbracht wird, ist für Deutschland noch wenig beleuchtet [10, 11]. Es stellt sich die Frage, ob die aufgrund der Mortalitätsreduktion zusätzlich gewonnenen Lebensjahre durch gute funktionelle Gesundheit oder erhöhte Beeinträchtigung gekennzeichnet sind.

Diese Frage wird seit geraumer Zeit diskutiert und führte zu unterschiedlichen Theorien des Verhältnisses von Lebenserwartung und verbleibender gesunder Lebenszeit. Die Kompressionstheorie postuliert, dass mit der Erhöhung der Lebenserwartung auch die Zahl der Jahre zunimmt, die in guter Gesundheit verbracht werden [12], während die Expansionstheorie die Gegenthese bildet und einen Anstieg der in Krankheit und Beeinträchtigung verbrachten Lebenszeit annimmt [13]. Gesunde Lebensjahre werden je nach Untersuchungsziel unterschiedlich definiert; unser Fokus liegt auf der funktionellen Gesundheit als Indikator. Unter funktioneller Gesundheit verstehen wir die Fähigkeit, in der Interaktion von gesundheitlichen Voraussetzungen und individuellen sowie umweltlichen Kontextfaktoren Tätigkeiten des täglichen Lebens (wie beispielsweise das Gehen längerer Strecken, Einkaufstaschen tragen, Treppensteigen oder Körperpflege) auszuführen [14, 15]. Sie ist auf individueller Ebene ein wichtiger Faktor für die Autonomie im Alter, bei unter 65-Jährigen auch für die Erwerbsfähigkeit, und beeinflusst Selbstständigkeit, Lebensqualität und Teilhabe wesentlich mit. Aus gesellschaftlicher Sicht ist eine gute funktionelle Gesundheit u. a. wichtig für die Vermeidung von Langzeitpflege.

Die bisherigen Befunde zu Kompression oder Expansion der Morbidität insbesondere in Bezug auf funktionelle Einschränkungen sind uneinheitlich. Mehrere Studien zeigen einen langfristigen Trend zu einem besseren Gesundheitszustand bei älteren Menschen [16], nicht aber bei den jüngeren Geburtsjahrgängen [8, 9, 17]. So wurden Verschlechterungen in der funktionellen Gesundheit unter Personen im mittleren und höheren Erwerbsalter gefunden [18]. Studien, die sich auf Aktivitäten des täglichen Lebens bei Älteren konzentrierten, fanden einen stabilen bzw. rückläufigen Trend des Anteils der Lebenszeit mit funktionellen Einschränkungen an der Lebenserwartung (z. B. [19]).

Zur Messung funktioneller Einschränkungen verwenden wir einerseits den standardisierten und international etablierten „Global Activity Limitation Indicator“ (GALI; [20–22]). Der GALI ermöglicht eine globale Erfassung der funktionellen Gesundheit, d. h. die Einschätzung der Auswirkungen gesundheitlicher Einschränkungen auf die Fähigkeit einer Person zur Ausführung von Alltagsaktivitäten. Dieser Indikator ist eine Schlüsselkomponente der EU-SILC-Erhebung (European Union Statistics on Income and Living Conditions) und wurde von mehreren Ländern übernommen, um Daten über Behinderungen und Funktionseinschränkungen zu sammeln [23]. Bei den GALI-Analysen beschränken wir uns auf die von starken funktionellen Einschränkungen freie Lebenserwartung und damit auf den Aspekt der substanziellen Beeinträchtigung der gesundheitlichen Lebensqualität.

Der Mobilität als spezifischem Bereich der funktionellen Gesundheit kommt im Alltag eine besondere Bedeutung zu. Sie beeinflusst die Lebensqualität wesentlich und sie hat einen hohen prognostischen Wert für Lebensqualität und Lebenserwartung. Einschränkungen der Mobilität sind mit schlechterem Wohlbefinden, mit Multimorbidität, Pflegebedürftigkeit und früherem Tod verbunden. Ältere Menschen selbst betrachten Mobilitätsverluste als einen wesentlichen Nachteil des Alterns [12].

Für die Messung von Mobilitätsbeeinträchtigungen wurden Items aus der Subskala „Körperliche Funktionsfähigkeit“ des 36-Item-Short-Form-Surveys (SF-36; [24, 25]) verwendet. Die Fähigkeit, *einen Treppenabsatz zu steigen*, wurde als Indikator gewählt, da sie eine grundlegende alltägliche Aktivität darstellt und zur Teilhabe beiträgt [26]. Treppensteigen setzt ein gewisses Maß an körperlicher Fitness voraus – Balance, Koordination sowie Muskelkraft, vor allem der Beinmuskulatur. Daher können Probleme beim Treppensteigen frühzeitig auf weitere funktionelle Einschränkungen hinweisen und sind nicht zuletzt eng mit der Unabhängigkeit im Alltag verbunden [27]. Probleme beim Treppensteigen sind außerdem mit dem Risiko von Stürzen und Sturzangst und damit einer eingeschränkten Unabhängigkeit im Alltag verbunden [28, 29]. Die Fähigkeit, *weiter als 1* *km zu Fuß zu gehen,* ist ein Maß dafür, wie gut aerobe Ausdauer, Muskelkraft und Beweglichkeit, insbesondere in den Beinen, sind. Auch dieser Indikator ist eng mit der Unabhängigkeit im täglichen Leben, beispielsweise beim Einkaufen, in der Arbeit oder bei anderen Aktivitäten verbunden. Das Gehen längerer Strecken kann ein Prädiktor für die künftige Entwicklung von Funktionseinschränkungen sein. Bei Älteren, die Schwierigkeiten hatten, eine längere Strecke zu gehen, wurden eine höhere Sterblichkeit, größere Funktionsdefizite, höhere Gesundheitskosten und mehr Krankenhausaufenthalte beobachtet [30]. Beide Indikatoren bilden unterschiedliche Aspekte der Mobilität ab: Während das Treppensteigen mehr auf koordinierte Bewegung, Balance und Muskelkraft fokussiert, steht das Gehen über längere Strecken in Verbindung mit aerober Ausdauer und Muskelkraft.

Wir untersuchen in dieser Studie, ob die Lebenserwartung ohne starke funktionelle und Mobilitätseinschränkungen ab 46 bzw. 65 Jahren zu- oder abgenommen hat und wie sich ihre Anteile an der Restlebenserwartung verändert haben.

## Methoden

### Daten

Datengrundlage der Studie ist der Deutsche Alterssurvey (DEAS), eine vom Bundesministerium für Familie, Senioren, Frauen und Jugend (BMFSFJ) geförderte Langzeitstudie des Deutschen Zentrums für Altersfragen (DZA) zur Lebenssituation und zu Alternsverläufen von Menschen in der zweiten Lebenshälfte. Der DEAS beruht auf bundesweit repräsentativen Befragungen im Quer- und Längsschnitt von Personen im Alter ab 40 Jahren [31]. Für die Analyse wurden Daten der Erhebungswellen 2008, 2014 sowie 2020/2021 verwendet. Bedingt durch die COVID-19-Pandemie wurden in der Welle 2020/2021 im Unterschied zu früheren Wellen nur Panelteilnehmende telefonisch und schriftlich befragt [32]. Daher sind in den Daten 2020/2021 keine 40- bis 45-jährigen Personen enthalten. Wegen zu geringer Fallzahlen haben wir außerdem Befragte im Alter ab 90 Jahren für die Prävalenzratenberechnung ausgeschlossen (Tab. 1, für Fallzahlen nach Altersgruppen siehe Onlinematerial Tab. A1).

**Table 1:** 

	2008	2014	2020/2021
*Studienteilnehmende (n)*	7413	9735	5329
*Altersgruppen (in %)*			
46–49 J.	13,3	12,2	9,2
50–54 J.	19,3	18,7	14,8
55–59 J.	13,4	13,8	18,3
60–64 J.	12,0	13,0	14,5
65–69 J.	14,8	11,0	11,6
70–74 J.	12,5	11,2	10,8
75–79 J.	8,0	10,9	6,9
80–84 J.	6,0	6,2	9,9
85–89 J.	0,7	3,1	4,0
*Geschlecht (in %)*			
Männlich	47,6	48,1	48,0
Weiblich	52,4	51,9	52,0
*GALI (in %)*			
Nicht (stark) eingeschränkt	90,2	87,1	88,7
Stark eingeschränkt	9,8	12,9	11,3
*1 Treppenabsatz steigen (in %)*			
Nicht eingeschränkt	86,5	85,1	85,1
Eingeschränkt	13,5	14,9	14,9
*>* *1* *km zu Fuß gehen (in %)*			
Nicht eingeschränkt	81,4	77,8	79,2
Eingeschränkt	18,6	22,2	20,8

*GALI* Global Activity Limitation Indicator

Das Einverständnis zur Teilnahme am DEAS wurde durch eine gemeinsame schriftliche Teilnahmebitte des DZA und Infas unter ausdrücklicher Zusicherung der Freiwilligkeit eingeholt. Die Befragten wurden vor Beginn der Studie schriftlich über die Ziele und Verfahren der Studie und die Einhaltung der Datenschutzbestimmungen aufgeklärt. Die Beantwortung aller Fragen erfolgte freiwillig.

Bei der Analyse der DEAS-Stichproben 2008, 2014 und 2020/2021 ergeben sich Limitationen aus dem Methodenwechsel von persönlichen zu telefonischen Interviews, aus der querschnittlichen Analyse von Gesamt- und Panelstichproben und aus den Besonderheiten der Befragung unter Pandemiebedingungen. Ein Teil der daraus resultierenden Probleme kann durch den Einsatz von Gewichtungsfaktoren ausgeglichen werden [33].

### Indikatoren der funktionellen Gesundheit

Der *GALI-Indikator* wurde im persönlichen Interview (2008, 2014) bzw. im telefonischen Interview (2020/2021) mit folgender Frage erfasst: „Waren Sie während der letzten 6 Monate oder länger bei Dingen, die man üblicherweise so tut, aus gesundheitlichen Gründen eingeschränkt?“ Antwortmöglichkeiten waren: „Ja, stark eingeschränkt“, „Ja, eingeschränkt“ und „Nein, nicht eingeschränkt“. Für die Berechnung der Disability Free Life Expectancy (DFLE) wurden jene Anteile der Befragten verwendet, die starke Einschränkungen berichtet haben.

Mobilitätseinschränkungen wurden durch 2 Indikatoren aus der Subskala Körperliche Funktionsfähigkeit des SF-36-Instruments [24] erfasst; sie basieren auf folgender Fragestellung: „Sind Sie durch Ihren derzeitigen Gesundheitszustand bei diesen Tätigkeiten stark eingeschränkt, etwas eingeschränkt oder überhaupt nicht eingeschränkt?“ Verwendet wurden die Items zu Einschränkungen beim Steigen eines Treppenabsatzes und beim Gehen von mehr als 1 km zu Fuß. In die Analyse wurden jeweils die Anteile der Befragten, die sich als „etwas“ oder „stark“ eingeschränkt bezeichneten, einbezogen.

### Mortalität

Für die Berechnung der Lebensjahre frei von funktionellen Einschränkungen und Mobilitätseinschränkungen wurden die Daten der Bevölkerungsstatistik des Statistischen Bundesamtes (mittlere Bevölkerung und Sterbefälle nach Geschlecht und Altersjahren für 2008, 2014 und 2020; [34, 35]) genutzt.

### Analysen

Die von funktionellen Einschränkungen freie Lebenserwartung (*Disability Free Life Expectancy – DFLE*) wurde mithilfe der Sullivan-Methode berechnet [36, 37]. Die Sullivan-Methode ermöglicht es, die Restlebenserwartung ab einem bestimmten Alter in „gesunde“ und „kranke“ Lebensjahre zu zerlegen. Basis für unsere Analyse bilden Sterbetafeln für Deutschland der Jahre 2008, 2014 und 2020, die auf den im Abschnitt Mortalität beschriebenen Populationsdaten basieren. Die Sterbetafeln wurden mithilfe der in [38] beschriebenen formaldemografischen Methoden der Sterbetafelanalyse separat für beide Geschlechter für die Jahre 2008, 2014 und 2020 berechnet.

Die Prävalenzen der funktionellen Einschränkungen wurden aufgrund der geringen Besetzung einzelner Altersjahre im DEAS für 5‑Jahres-Altersgruppen berechnet. Die Mortalitätsdaten des Statistischen Bundesamtes liegen dagegen für jedes Altersjahr vor und bilden die Basis für die Restlebenserwartung ab diesem Altersjahr. Wir haben die DFLE-Werte für 2 Altersjahre analysiert – für die 46-Jährigen, um die gesamte zweite Lebenshälfte in den Blick zu nehmen, und für die 65-Jährigen als Repräsentanten des „jungen“ Alters im Übergang in den Ruhestand. Die gewichteten altersspezifischen Prävalenzanteile der verwendeten 3 Indikatoren der funktionellen Einschränkungen wurden auf Basis der DEAS-Wellen 2008, 2014 sowie 2020/2021 getrennt nach Geschlecht und in 5‑Jahres-Altersgruppen geschätzt. Basierend auf diesen Anteilen wurden die im jeweiligen Alter noch zu erwartenden Lebensjahre in Jahre mit und ohne funktionelle Einschränkungen eingeteilt. Darüber hinaus berechneten wir den sogenannten Health Ratio (HR) als Quotient der im jeweiligen Alter noch zu erwartenden Lebensjahre ohne funktionelle Einschränkungen und der (Rest‑)Lebenserwartung [37, 39]. Dies ermöglichte eine Einschätzung der absoluten und relativen Veränderungen der Lebenszeit mit und ohne funktionelle Einschränkungen für die deutsche Bevölkerung ab dem mittleren Lebensalter. Die Analysen zur DFLE in Jahren sowie in Prozentanteilen an der Lebenserwartung wurden mithilfe eines Templates des „European Health and Life Expectancy Information Systems“ (EHLEIS) durchgeführt [37]. Wir fokussieren unsere Analysen auf 2 DFLE-Werte – auf die einschränkungsfreie Lebenszeit ab dem Alter 46 und ab dem Alter 65. Das Signifikanzniveau für die Analysen wurde auf *p* < 0,05 festgelegt.

## Ergebnisse

### Lebenserwartung ohne starke funktionelle Einschränkungen (Indikator DFLE_GALI_)

**Figure 1:**
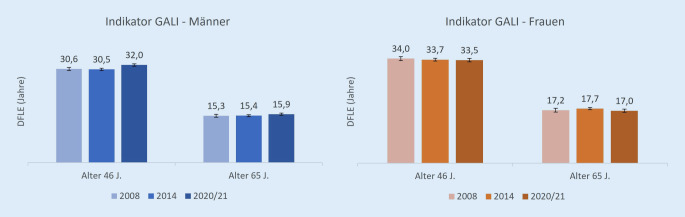

Die Lebenserwartung ohne starke funktionelle Einschränkungen in Jahren (DFLE_GALI_) betrug im Jahr 2020/2021 bei 46-jährigen Männern 32,0 Jahre und bei gleichaltrigen Frauen 33,5 Jahre (Abb. 1). 65-jährige Männer und Frauen können noch 15,9 bzw. 17,0 Jahre DFLE_GALI_ erwarten. Im Vergleich zu Männern wiesen Frauen damit in 2020/2021 in beiden Altersgruppen ein signifikant höheres Niveau der DFLE_GALI_ in Jahren auf. Ihr HR_GALI_, also der prozentuale Anteil der DFLE_GALI_ im Alter x an der Restlebenserwartung im Alter x, ist jedoch niedriger als bei Männern – um 6,1 Prozentpunkte bei 46-Jährigen und um 7,8 Prozentpunkte bei 65-Jährigen.

Die zeitliche Entwicklung zeigt insbesondere für Männer ab dem mittleren Alter einen positiven Trend (Abb. 1 und Tab. 2): Zwischen 2008 und 2020/2021 stieg die DFLE_GALI_ bei *46-jährigen Männern* von 30,6 Jahren auf 32,0 Jahre. Der HR_GALI_ erhöhte sich für 46-jährige Männer zwischen 2014 und 2020/2021 signifikant von 87,2 % auf 91,3 %. Für *65-jährige Männer* wurde keine signifikante absolute Verbesserung der DFLE_GALI_ festgestellt. Der HR_GALI_ stieg zwischen 2014 und 2020/2021 an, allerdings nicht signifikant.

**Table 2:** 

Geschlecht	Alter	HR in % (95 %-KI)		
		2008	2014	2020/2021
*Starke funktionelle Einschränkungen (GALI)*				
Männer	46 J.	89,6 (88,2–91,1)	87,2 (86,1–88,2)	91,3 (90,2–92,5)
	65 J.	87,3 (84,4–90,2)	84,6 (82,8–86,5)	88,3 (86,2–90,3)
Frauen	46 J.	88,1 (86,5–89,7)	85,4 (84,3–86,5)	85,2 (83,8–86,6)
	65 J.	83,4 (80,4–86,5)	83,1 (81,3–84,9)	80,5 (78,1–82,9)
*Steigen eines Treppenabsatzes*				
Männer	46 J.	86,9 (85,2–88,6)	85,3 (84,2–86,3)	88,1 (86,8–89,4)
	65 J.	78,6 (75,1–82,1)	76,4 (74,4–78,4)	81,2 (78,7–83,7)
Frauen	46 J.	80,3 (78,5–82,2)	80,0 (78,8–81,2)	81,0 (79,6–82,4)
	65 J.	68,2 (64,7–71,8)	69,2 (67,1–71,3)	68,4 (65,8–71,0)
*Mehr als 1* *km gehen*				
Männer	46 J.	81,7 (80,0–83,5)	77,7 (76,5–78,9)	81,3 (79,8–82,8)
	65 J.	70,8 (67,1–74,4)	67,5 (65,4–69,6)	69,8 (66,9–72,6)
Frauen	46 J.	72,9 (71,1–74,7)	71,7 (70,5–72,9)	74,9 (73,4–76,5)
	65 J.	57,0 (53,7–60,2)	58,4 (56,3–60,4)	61,2 (58,6–63,8)

Daten Prävalenzanteile: Deutscher Alterssurvey (DEAS) 2008, 2014, 2020/2021, gewichtet; Daten Mortalität: Statistisches Bundesamt, Genesis online. Eigene Berechnungen

*GALI* Global Activity Limitation Indicator, *KI* Konfidenzintervall

Die DFLE_GALI_ in Jahren bei *46-jährigen Frauen* veränderte sich zwischen 2008 und 2020/2021 nicht signifikant. Der HR_GALI_ zeigte jedoch eine negative Tendenz (Tab. 2): Er sank von 88,1 % (2008) auf 85,2 % (2020/21). Die DFLE_GALI_ *bei 65-jährigen Frauen *nahm zwischen 2014 und 2020/2021 sogar ab, von 17,7 Jahren auf 17,0 Jahre. Der HR_GALI_ war bei *65-jährigen Frauen* zwar ebenfalls rückläufig, jedoch nicht signifikant.

### Lebenserwartung ohne Einschränkungen beim Steigen eines Treppenabsatzes (Indikator DFLE_Treppe_)

**Figure 2:**
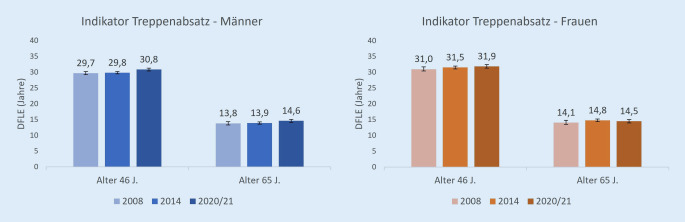

46-jährige Männer hatten im Jahr 2020/2021 mit einer DFLE_Treppe_ von 30,8 Jahren einen signifikant niedrigeren Wert als Frauen mit 31,9 Jahren. Bei 65-Jährigen betrug dieser Wert 14,6 Jahre für Männer und 14,5 Jahre für Frauen, wobei die Differenz nicht signifikant war (Abb. 2).

Die DFLE_Treppe_ zeigt bei Männern im Alter 46 eine positive Entwicklung, die auch hier vorrangig aus dem Anstieg zwischen 2014 und 2020/2021 resultiert: Zwischen 2008 und 2020/2021 stieg der Wert dieses Indikators bei *46-jährigen Männern* von 29,7 Jahren auf 30,8 Jahre (Abb. 2). Der HR_Treppe_ änderte sich in diesem Zeitraum dagegen nicht signifikant (Tab. 2). Bei *65-jährigen Männern* änderten sich die Werte der DFLE_Treppe_ und der HR_Treppe_ im betrachteten Zeitraum nicht signifikant.

Im Gegensatz dazu zeigt dieser Indikator weder bei *Frauen* mittleren (46 Jahre) noch höheren Alters (65 Jahre) eine signifikante Veränderung über den betrachteten Zeitraum. Dies gilt sowohl für die DFLE_Treppe_ in Jahren als auch für den HR_Treppe_.

### Lebenserwartung ohne Einschränkungen beim Gehen von mehr als 1 km (Indikator DFLE_km_)

**Figure 3:**
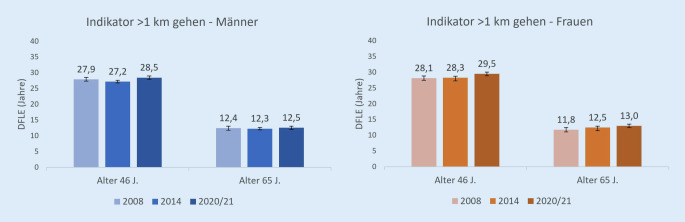

46-jährige Männer hatten im Jahr 2020/2021 eine DFLE_km_ von 28,5 Jahren und damit einen tendenziell niedrigeren Wert als Frauen mit 29,5 Jahren. Bei 65-Jährigen lag dieser Wert bei 12,5 Jahren (Männer) bzw. 13,0 Jahren (Frauen), die Differenz ist nicht signifikant (Abb. 3).

Über den Gesamtzeitraum von 2008 bis 2020/2021 hat sich die DFLE_km_ für 46-jährige Männer nicht signifikant verändert. Zwischen 2014 und 2020/2021 stieg sie jedoch signifikant von 27,2 Jahren auf 28,5 Jahre. Bei 65-jährigen Männern zeigten sich hingegen keine signifikanten absoluten Veränderungen (Abb. 3 und Tab. 2). Der HR_km_ blieb sowohl bei 46-jährigen als auch 65-jährigen Männern annährend konstant über die Zeit.

Von 2008 bis 2020/2021 erhöhte sich die DFLE_km_ für 46-jährige Frauen von 28,1 Jahren auf 29,5 Jahre. Der HR_km_ stieg zwischen 2014 und 2020/2021 signifikant von 71,7 % auf 74,9 %. Auch für ältere Frauen stieg die DFLE_km_ zwischen 2008 und 2020/2021. 65-jährige Frauen hatten 2008 eine DFLE_km_ von 11,8 Jahren und 2020/2021 von 13,0 Jahren. Dies führte jedoch nicht zu einem signifikanten Anstieg des HR_km_ bei älteren Frauen.

## Diskussion

Ziel dieser Studie waren empirische Erkenntnisse zu der Frage, ob es in den Jahren seit 2008 in Deutschland bei der funktionellen Gesundheit von Erwachsenen ab dem mittleren und höheren Alter zu einer Kompression oder Expansion der verbleibenden Lebenszeit mit funktionellen Einschränkungen gekommen ist. Zu diesem Zweck wurden die Veränderungen der DFLE in Jahren und des HR für 3 Indikatoren der funktionellen Gesundheit bei Männern und Frauen ab dem Alter 46 und 65 für die Jahre 2008, 2014 und 2020/2021 analysiert. Die aktuellen Daten aus der DEAS-Welle 2020/2021 legen nahe, dass die DFLE (gemessen in Jahren) bei Frauen höher ist als bei Männern, aufgrund der insgesamt höheren Lebenserwartung der Frauen.

Unsere Analysen lassen keine eindeutige Antwort auf die Frage nach Morbiditätskompression oder -expansion zu. Wir sehen je nach Indikator, Alter und Geschlecht unterschiedliche Trends. Die Heterogenität in der Morbiditätsentwicklung, die gleichzeitig Kompression und Expansion der Morbidität einschließt, spiegelt die Komplexität dieses Themas wider und wird auch von anderen Studien, wie [19], unterstützt. Für 46-Jährige zeigen unsere Befunde zum Teil stagnierende Tendenzen. Dieser Trend wurde in den vergangenen Jahren u. a. von [18, 40–42] und [43] berichtet. Im höheren Alter (65 Jahre) entwickelte sich die einschränkungsfreie Lebenserwartung zwischen den Geschlechtern zum Teil unterschiedlich. Bezüglich der starken funktionellen Einschränkungen sehen wir bei älteren Männern einen Trend in Richtung Kompression der Morbidität, bei den älteren Frauen dagegen eher Stagnation. Die Mobilität stagnierte im betrachteten Zeitraum bei den Älteren, mit Ausnahme des Treppensteigens bei 65-jährigen Männern – bei diesem Indikator liefern die Daten Hinweise auf Kompression der Morbidität. Diese Entwicklung von Funktionseinschränkungen ist u. a. auch von [44] berichtet worden.

Eine wichtige Erkenntnis unserer Studie ist, dass Frauen gegenwärtig möglicherweise weniger von der allgemeinen Gesundheitsentwicklung profitieren als Männer. Ab dem mittleren Alter (46 Jahre) deuten die Daten auf eine mögliche Expansion der Morbidität bei Frauen hin, insbesondere bei starken funktionellen Einschränkungen, während es für Männer Anzeichen einer Kompression gibt. Diese Tendenz, die auch in früheren Studien belegt wurde [44, 45], zeigt sich in stagnierenden oder rückläufigen HR-Werten bei Frauen für einige Indikatoren. Für die Ursachen dieser unterschiedlichen Entwicklung bei Männern und Frauen gibt es lediglich erste Anhaltspunkte: Einige Risikofaktoren für funktionelle Einschränkungen scheinen bei Frauen häufiger aufzutreten, darunter Arthritis, depressive Symptome, sturzbedingte Frakturen sowie Alzheimer-Krankheit und verwandte Demenzen. Daneben werden auch eine häufigere körperliche Inaktivität von Frauen und fehlende wirtschaftliche Ressourcen für den Ausgleich funktioneller Defizite als Ursachen diskutiert [44]. Unsere Ergebnisse ordnen sich damit in den Kontext ähnlicher Studien ein und konnten diese mit Indikatoren für funktionelle Gesundheit des DEAS bestätigen.

### Limitationen

Der DEAS ist eine bevölkerungsrepräsentative Erhebung mit Basis- und Panelstichproben. Aufgrund der Pandemiesituation wurde der DEAS 2020/2021 erstmals mit einer telefonischen Befragung erhoben. Dies und die besondere Situation der Pandemiezeit könnten die Vergleichbarkeit zu den älteren Wellen eingeschränkt haben. Ähnliches gilt für die Stichproben: Für die Jahre 2008 und 2014 wurden die Gesamtstichproben der Erhebungen (Basis- und Panelstichprobe) und für 2020/2021 eine reine Panelstichprobe ausgewertet. Die Zusammensetzung der Befragung im Jahr 2020/2021 aus ausschließlich Panelbefragten könnte eine gewisse Überschätzung der zum Teil gefundenen Anstiege der DFLE zwischen 2008 und 2020/2021 begünstigt haben. Durch die Anwendung der Gewichtungsfaktoren wurden diese Stichprobenverzerrungen jedoch soweit wie möglich ausgeglichen. Die Besonderheiten, die aus Moduswechsel und Stichprobenzusammensetzung resultieren, sind bei der Interpretation der Ergebnisse zu beachten. Mit weitergehenden Analysen werden derzeit diese Auswirkungen untersucht [46]. Die Entwicklung der DFLE in der deutschen Bevölkerung sollte weiter beobachtet werden, um die Evidenz zu den langzeitlichen Zeittrends zu stärken. Bei der Interpretation ist außerdem zu beachten, dass im DEAS nur Daten von Personen in Privathaushalten erhoben werden. Eine weitere Limitation ist, dass stark gesundheitlich eingeschränkte Personen häufig in geringerem Umfang an Surveys teilnehmen und Personen in institutionalisierten Einrichtungen ebenfalls nicht im Sample enthalten sind. Eine weitere Einschränkung besteht darin, dass die Daten zu den funktionellen Einschränkungen auf Selbstberichten der Befragten, nicht auf Messungen, beruhen.

### Schlussfolgerungen

Die Ergebnisse unserer Studie liefern wichtige Einblicke in die Entwicklung der funktionellen Gesundheit von Erwachsenen ab dem mittleren und höheren Erwachsenenalter in Deutschland. Die Vielfalt in der Morbiditätsentwicklung, die sowohl Kompression als auch Expansion der Morbidität und funktionellen Einschränkung umfasst, unterstreicht die Komplexität dieses Forschungsgebiets.

Angesichts der Entwicklung der geschlechtsspezifischen Unterschiede in der funktionellen Gesundheit sollten gesundheitspolitische Maßnahmen gezielt auf die Bedürfnisse von Frauen und Männern eingehen. Insbesondere bei der Prävention schwerwiegender funktioneller Einschränkungen im mittleren Erwachsenenalter könnte eine verstärkte Förderung von Gesundheitsprogrammen für Frauen erforderlich sein, um einer potenziellen Expansion der Morbidität entgegenzuwirken. Eine gendersensible Gesundheitsförderung könnte beispielsweise bedeuten, dass Programme zur Förderung von körperlicher Aktivität oder Rehabilitation spezifische Trainingsmethoden oder Settings für Frauen und Männer anbieten. Außerdem ist es wichtig, die altersspezifischen Unterschiede in der Morbiditätsentwicklung zu berücksichtigen. Im mittleren Erwachsenenalter sind möglicherweise präventive Maßnahmen zur Verlangsamung von funktionellen Einschränkungen erforderlich, während im höheren Alter eine altersangepasste Herangehensweise erforderlich ist, die auch den Geschlechtsunterschieden Rechnung trägt.

Gesundheitspolitische Strategien sollten geschlechtsspezifische Perspektiven integrieren, um sicherzustellen, dass die Bedürfnisse von Frauen und Männern gleichermaßen berücksichtigt werden. Dies könnte die Entwicklung von gezielten Gesundheitsprogrammen, Forschungsförderung und präventiven Maßnahmen einschließen.

### Supplementary Information




