# Supplementary material for: How has life expectancy without functional limitations developed in Germany? An analysis with data from the German Ageing Survey (DEAS)
Source: Bundesgesundheitsblatt Gesundheitsforschung Gesundheitsschutz. 2024 Apr 22;67(5):564–71. [Article in German] doi: 10.1007/s00103-024-03875-9 (PMC11093863; doi:10.1007/s00103-024-03875-9)
Supplement: Supplementary file 1 [file 103_2024_3875_MOESM1_ESM.pdf]

Onlinematerial

zum Artikel: „Wie hat sich die Lebenserwartung ohne funktionelle Einschränkungen in Deutschland entwickelt? Eine Analyse mit Daten des Deutschen Alterssurveys (DEAS)“

Bundesgesundheitsblatt Ausgabe 5/2024

Tabelle A1: DEAS-Fallzahlen für 2008, 2014 und 2020/21 nach Altersgruppen und Geschlecht

**Altersgruppe**	**DEAS 2008**		**DEAS 2014**		**DEAS 2020/21**	
	Männer	Frauen	Männer	Frauen	Männer	Frauen
**46-49J.**	511	475	627	567	249	239
**50-54J.**	783	652	980	847	421	370
**55-59J.**	530	465	671	668	473	503
**60-64J.**	422	463	670	597	402	373
**65-69J.**	599	500	538	532	341	277
**70-74J.**	444	479	581	508	254	320
**75-79J.**	280	309	474	584	169	198
**80-84J.**	190	256	247	347	232	296
**85-89J.**	20	34	115	180	76	134
